# The Outlook of Medicine and Surgery. No. 3

**Published:** 1883-08

**Authors:** J. Mc. F. Gaston

**Affiliations:** Campinas, S. Paulo, Brazil


### Original.

### THE OUTLOOK OF MEDICINE AND SURGERY.

Number 3.

BY J. McF. GASTON, M.D., CAMPINAS, S. PAULO, BRAZIL.

The clinical phase of medical literature includes not only the proper application of means of relief, but all that knowledge of the capacities of the human organization upon which the effect of curative agencies depends. A practitioner may be engaged for a series of years in the treatment of diseases without that scrutiny and close examination of the nature of his cases that constitutes real experience in his profession. His observation in regard to the source of the derangement of vital organs, and the complications of the disturbance of their functions by mutual reactions, must be carried into all the details of the development of the different disorders of the animal economy, and if he contents himself with a superficial attention to the symptoms of disease, he is sure to overlook indications essential to the proper use of remedies. Clinical study of disease is therefore of the highest importance to success in practice and the beginning, progress and result of different forms of disease, should not only be noted, but recorded by everyone who would profit by his observation, or who would make his experience useful to others. There are separate spheres of duty for different members of the medical profession that should respectively employ their individual energies, and yet all are so related as to reciprocally aid each other, while they promote the grand total of progress in practical results by mutual co-operation and assistance. One physician may have a peculiar capacity for the recognition of the seat of disorders, and hence excel in diagnosis; another may be endowed with more than ordinary tact in determining the nature of wholesale complex modifications of diseased action; while a third will have a greater insight of the special means of cure adapted to individual cases. But the great desideratum for each is to seek as far as may be practicable a thorough comprehension of all that pertains to his patient, and not rest satisfied to leave anything hidden or obscure in the etiology or pathology of the case under examination. Not that everything should be entirely rid of doubt before resorting to any measure of treatment, for enough may be elucidated to determine upon the use of a certain class of remedies, which may be applied as a means of further elucidation of the origin of the trouble, and thus become in some form a tentative or explanatory application. As a simple illustration, a case of nausea is presented and the physician is at a loss as to the real cause, when he prescribes an emetic, and finds the contents of the stomach have undergone fermentation decomposition, by the dislodgment of which the patient is entirely relieved. It is the only requisite to use means to obviate the recurrence of such a condition, to effect a cure; whereas, if such medication had been used at the outset, as is usually adopted for correcting nausea, the result could not have been so satisfactory. Yet, notwithstanding the necessity for tentative practice occasionally, the general rule holds good, that all the symptoms should be noted as a preliminary to treatment. The old saying that "practice makes perfect" holds only with those who attend properly to the execution of a task in any department of service, and more especially does it devolve upon the physician or surgeon to scrutinize each step in the performance of his work, that improvement may crown his undertaking. If he be a mere routinist and follow in the wake of some one who has gone before, without understanding the principles involved in his use of remedies, it is most likely that he will only be confirmed in the errors of the past. But if he investigates for himself, and with all the lights before him seeks to discern a reason for all that is done, his observation yields the legitimate fruits of experience, and he makes a contribution of value to clinical statistics. He who would derive real advantage from experience in the practice of medicine and surgery, must bring to the work an intelligent comprehension of all that is available in his field of duty, and "strive for the mark of the high calling that is set before him." In this undertaking, as in most of the enterprises that claim the energies of man, zeal without knowlodge avails little, and study must accompany clinical observation to secure a good result in practice. There is a charm about success that insures success in the subsequent efforts of an individual, but the first element of success in any branch of service is to master the difficulties in the way by laborious and assiduous preparation for the work.

In the therapeutic applications of the first physicians all was necessarily experimental, and the properties of drugs came only to be recognized with their repetition in similar cases; but such varied uses have been since made of most of the standard articles of the materia medica that the effects of given doses in given cases can be anticipated with a probability that approaches certainty. Under such circumstances the knowledge of the data accumulated by the experience of those who have gone before places the practitioner of the present day upon a decided vantage ground, and if he battles with disease understandingly, and turns to account the armory that is provided for his use, he ought to secure more satisfactory results than was available to his predecessors.

The well-defined limits of the known is, however, bounded by a circle of doubtful measures, and this surrounded again by the great unknown, so that there is a wild field yet for exploration and for careful investigation. Clinical observation thus presents a sphere for judicious experiment that is very attractive to the enterprising student of medical science, and as we have reaped the fruits of past observation, it becomes us to map out what has been obtained with accuracy in the hitherto doubtful circle, and to push forward our investigations into the unknown border land. Others may profit by our failures, if we have no grand discoveries to reward our efforts, and truthful statistics are useful beacons, if not guides, for all who come after us. There is too great proneness on the part of practitioners to herald cures, without noting their shortcomings, and the only medical journal that has opened a column for these found few to occupy it.

The therapeutic principle involved in the operation of a given article of the materia medica in different disorders is not ascertained with accuracy, and much difference of opinion prevails as to thc precise element of the organisms that is effected primarily by the action of medical agents. Whether the specific quality of vomiting, purging, etc., depends upon a special determination of the medicine to certain organs, or is the result of a general impression upon the organization, thence distributed so as to influence the functions of the organs, does not belong to the class of fixed facts; and although certain results may be uniformly anticipated from prescribed quantities of different drugs, the why and wherefore are not clearly established. The great cardinal distinction recognized in the classification of the materia medica is based upon the effects of medication, and indicates nothing as to the mode of producing the result. It is held by some that medicines are invariably absorbed and eaten into the circulation of the blood, thus propagating their influence to the various parts, while others claim that this circuitous channel is not requisite for the properties of the article to be developed upon the nervous system, and transmitted through the nerve centres to the different organs of the body. A corrolation between the ultimate ramifactions of the nerves and the minute capillary blood vessels, favors the view that local application of medicines may affect remote parts without passing through the entire circuit of the sanguiferous circulation; and this is explained by the comparatively recent discovery of a ganglionic property of the cutaneous and other peripheral distributions of the nerve fibrils. There is no doubt but that some medicines require to be absorbed to produce their effects, while others act primarily upon the nervous system through the capillaries. It is known by all that the most direct route for medication is through the subcutaneous analar tissues, and more speedy effects are developed by application in this form than by any other channel of introduction for nervine properties, while those having properties distinct from this class operate appropriate results upon the system. The mucous membrane also offered a ready means of introducing medication, and the nervous tissues serve in like manner as an inlet to medicinal solutions, the results being manifested subsequently upon the general organization. Articles administered by the stomach have to undergo as a rule a species of digestion before they can produce their effects, yet there are some classes of medicine which operate immediately on the coats of this organ, and a stimulant or sedative influence is propagated directly through its nerves to the whole organism. It should not be understood from this statement that all medicines that go into the stomach are limited to these modes of action, as it is ascertained that many agents pass from the stomach with their properties unchanged, and are even detected in the different secretions and excretions of the body, modifying to a greater or less extent the parts with which they came in contact on their route. The external or superficial application of medicinal agents produces local or general effects that are very notable, yet more tardy than by other processes, and are only resorted to in compensation of impediments to other more effective means of treatment. Another and diverse end is attained by the class of cutaneous irritants, whose influence belongs to the important sphere of revulsions, and depends upon the sympathies of the nervous distribution for the striking results.

[To be continued.]

